# Resident duty hours in Canada: a survey and national statement

**DOI:** 10.1186/1472-6920-14-S1-S9

**Published:** 2014-12-11

**Authors:** Mark F Masterson, Pankaj Shrichand, Jerry M Maniate

**Affiliations:** 1 Canadian Association of Internes and Residents, Ottawa, Canada; 2 Department of Anesthesiology, Pharmacology and Therapeutics, University of British Columbia, Vancouver, Canada; 3 Department of Medicine, St. Joseph’s Health Centre, University of Toronto, and Wilson Centre for Research in Education, Faculty of Medicine, University of Toronto, Toronto, Canada

## Abstract

Physicians in general, and residents in particular, are adapting to duty schedules in which they have fewer continuous work hours; however, there are no Canadian guidelines on duty hours restrictions. To better inform resident duty hour policy in Canada, we set out to prepare a set of recommendations that would draw upon evidence reported in the literature and reflect the experiences of resident members of the Canadian Association of Internes and Residents (CAIR). A survey was prepared and distributed electronically to all resident members of CAIR. A total of 1796 eligible residents participated in the survey. Of those who responded, 38% (601) reported that they felt they could safely provide care for up to 16 continuous hours, and 20% (315) said that 12 continuous hours was the maximum period during which they could safely provide care (n = 1592). Eighty-two percent (1316) reported their perception that the quality of care they had provided suffered because of the number of consecutive hours worked (n = 1598). Only 52% (830) had received training in handover (n = 1594); those who had received such training reported that it was commonly provided through informal modelling. On the basis of these data and the existing literature, CAIR recommends that resident duty hours be managed in a way that does not endanger the health of residents or patients; does not impair education; is flexible; and does not violate ethical or legal standards. Further, residents should be formally trained in handover skills and alternative duty hour models.

## Introduction

Increasing attention has been paid in recent years to resident fatigue caused by long consecutive hours of work and its implications for patient safety and the safety, well-being, and education of residents [1-3]. Recent editorials have highlighted the effects of sleep deprivation on all physicians, rather than only physicians-in-training [4,5].

Although various resident duty hours are regulated in the United States and in member countries of the European Union [6,7], there are no national Canadian guidelines for resident duty hour restriction. Work hours are negotiated individually within each province. Quebec has recently negotiated a limit of 16 continuous hours of work [8]. Manitoba and the Maritime provinces have work-hour limits of 89 and 90 hours per week, respectively [9,10], and several provinces limit consecutive work hours to 24, with adequate handover. Protracted periods of sleeplessness are common in Canadian training, and there is little experience with alternative scheduling models [11].

To better inform resident duty hour policy in Canada, we set out to prepare a set of recommendations that would draw upon evidence reported in the literature and reflect the experiences of resident members of the Canadian Association of Internes and Residents (CAIR).

## The evidence on resident fatigue

A literature search was performed by one of the authors (PS) in conjunction with CAIR to identify literature that could inform the Canadian dialogue. Sources included a literature search used in the preparation of previous CAIR work [12], articles referred to us by provincial house staff organizations, residents with expertise in medical education, and PubMed and Google Scholar searches using combinations of the search terms “resident,” “physician trainee,” “duty hours,” “work hours,” “fatigue,” and “Canada.” “Related articles” links as provided by the respective search engines were also pursued. Key articles were selected and summarized by PS in conjunction with the Advocacy and Policy Committee of CAIR; their findings are summarized in Table 1.

**Table 1:** Selected summary of findings of the effects of resident fatigue

Patient safety/care	Resident safety/wellness	Cognition/ability to learn
Of all resident errors, 5%-36% are caused by fatigue [13].	First year residents reported a higher rate of injury (exposure to contaminated bodily fluids, percutaneous injuries) when fatigued [14].	The rate of falling asleep during lectures rose significantly with the number of extended-duration shifts worked in a given month [15].

For residents working more than 80 hours a week, the odds ratio of having a patient in the last week who experienced an adverse event was 1:8 [16].	Residents were most exposed to blood-borne pathogens through needle punctures or cuts during overnight work periods [17].	Staff physicians who were on call overnight had reduced performance in standard cognitive performance tests [18].

A randomized controlled trial of duty hour reduction found a significantly higher occurrence of serious medical errors with longer duty hours and less sleep [19].	Survey findings showed that residents were 2.3 times more likely to be involved in a motor vehicle crash after working an average of 32-hour shifts [20].	One night without sleep reduced third-year residents’ performance on tests to the level of a first-year resident [21].

Residents made twice as many errors reading ECGs after being awake for 24 hours [22].	Residents working shifts of more than 24 hours were at greatly increased risk of an occupational injury, a vehicle crash after work, and serious or fatal medical errors [23].	Being awake for more than 16 consecutive hours had an effect on cognitive performance equivalent to a 0.05%-0.10% blood alcohol concentration [24-27].

Surgical residents who had been awake all night made 20% more errors in completing a simulated laparoscopic surgical task than those who had had a full night’s sleep [28].	A survey of first- and second- year residents found that those who reported obtaining less than five hours of sleep per night were more likely to report increased use of alcohol and medications [29].	

Residents made more technical errors in simulated laparoscopic surgical skills after being awake through the night [30].		

### The CAIR duty hour survey

CAIR surveyed its membership on issues related to work hours. Questions were prepared on the basis of the literature summary described above. A CAIR committee with representation from all residency years and from medical, surgical, and family medicine specialties identified gaps in the existing data in relation to the Canadian context and designed survey questions to assess these areas. These gaps included information about Canadian residents’ current work hours, perceptions of the effect of work hours on patient safety, and training in patient safety and handover. The questions were then reviewed by the CAIR Board of Directors.

## Methods

### Survey administration

Resident members of CAIR or of any affiliated provincial housestaff organization (PHO) during the 2010-2011 academic year were eligible to participate in the survey. CAIR represents 7894 residents in Canada in the various provinces. These residents are represented locally by their PHOs, which are the Professional Association of Residents of British Columbia (PAR-BC), the Professional Association of Resident Physicians of Alberta (PARA), the Professional Association of Internes and Residents of Saskatchewan (PAIRS), the Professional Association of Residents and Interns of Manitoba (PARIM), the Professional Association of Internes and Residents of Ontario (PAIRO), the Professional Association of Residents in the Maritime Provinces (PARI-MP), and the Professional Association of Internes and Residents of Newfoundland (PAIRN). Residents in Quebec were not included as they are not members of CAIR.

An invitation to participate in the survey was sent by email to residents through their respective PHOs beginning in the last week of June 2011; the invitation was open for five weeks, that is, until the end of July 2011. Two reminder notices were sent during that time. The invitation included a link to an online survey tool. The survey included basic demographic questions; responses were anonymized. An incentive prize was offered by random draw. The first page of the survey was an informed consent form.

### Data analysis

The survey data were collected using an online platform, Survey Monkey, and were analyzed using descriptive statistics in Excel.

## Results

We invited 7894 residents to participate in the survey. Of the 1831 individuals who responded, 1796 were eligible for inclusion (a response rate of 23%). The 35 people who were not eligible indicated that they were not residents or members of a PHO. Respondents included residents in all years of training from medical schools in Canada outside of Quebec; 55% were women. See additional file 1 for respondent demographics.

**Figure 1:**
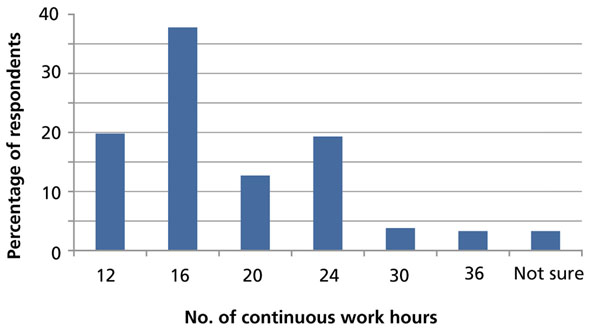
**Residents’ view of maximum continuous work hours.** In response to the question, “Up to how many continuous hours of work do you feel that you are able to consistently provide safe, high quality patient care,” residents were asked to choose 12, 16, 20, 24, 30, or 36 hours, or “not sure”.

Residents reported working a median of 65 hours per week (interquartile range 60-80). Of those who responded to this question (n = 1592), 38% (601) reported that they felt they could “consistently provide safe, high quality patient care” for up to 16 continuous hours, and 20% (315) said they felt that 12 continuous hours was the maximum period during which they could safely provide care (Figure 1).

**Figure 2:**
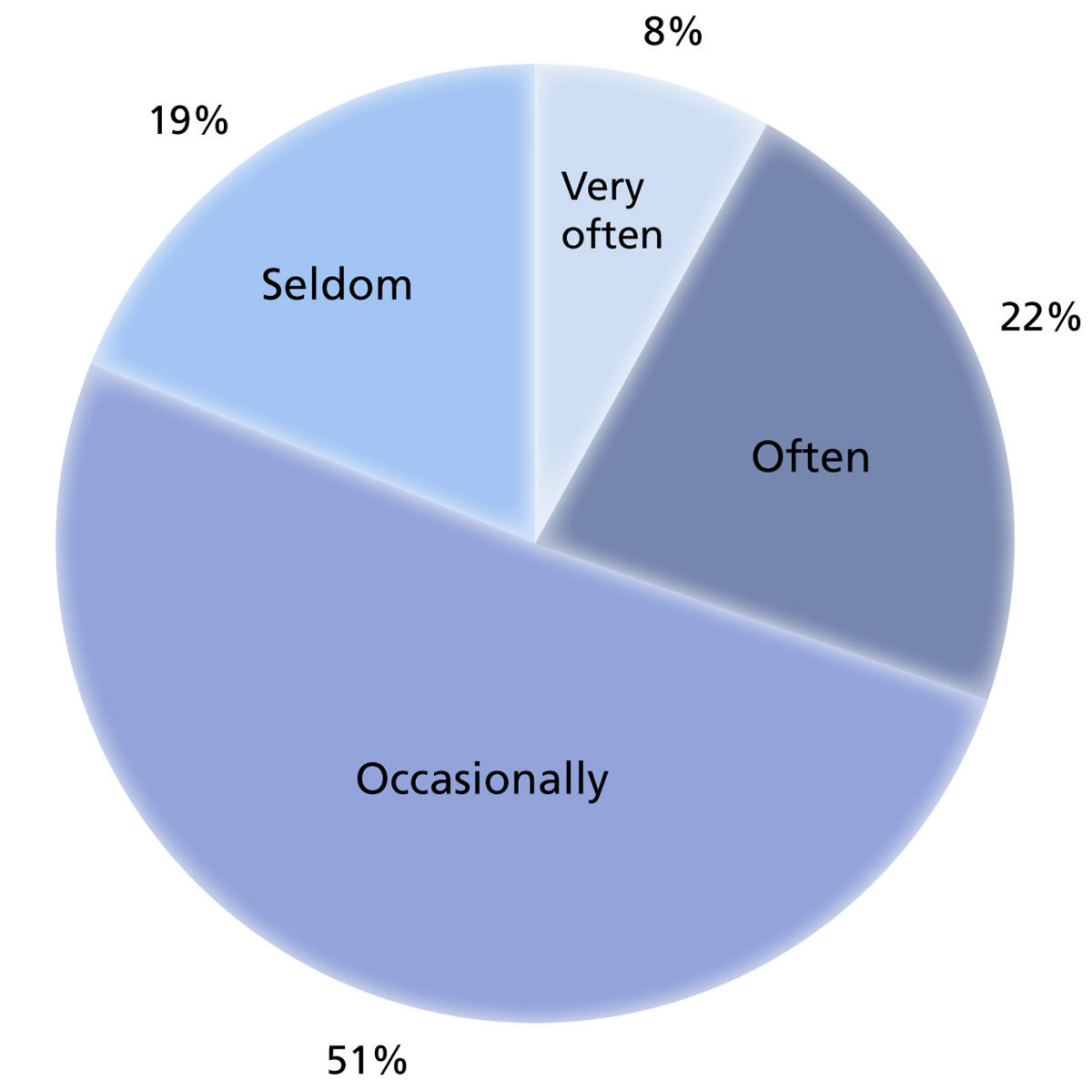
**Reported impact of work hours on patient care.** Residents were asked how often felt that the quality of the care they provided was compromised by the number of consecutive hours they had worked.

Eighty-two percent (1316) of respondents (n = 1598) answered “yes” to the question, “Have you ever felt that the quality of care you provide patients has been compromised by the number of consecutive hours you have worked?” Those who replied “yes” were then asked to indicate how frequently they felt this happened; 30% (397) reported that they felt the quality of care they provided was compromised “Often” or “Very often,” while only 19% (244) responded that this “Seldom” happened (Figure 2).

To the question, “Does your program provide training on how work hours affect patient and resident safety?”, 20% (312) of respondents (n = 1597) responded in the affirmative, 56% (893) in the negative, and 25% (392) were unsure. Of the 20% who reported that their program did offer such training, only 58% indicated that they were able to participate.

To the question, “Have you received training during your residency on how to transfer patient care to another provider (i.e., handover)?”, 52% (830) responded “yes” (n = 1594). Of the 48% who had not received training in handover, 24% responded that they felt this absence of training had adversely affected their ability to provide safe, high-quality care to their patients, 32% said they were unsure, and 44% said they felt this did not affect their ability to provide safe, high-quality care.

**Figure 3:**
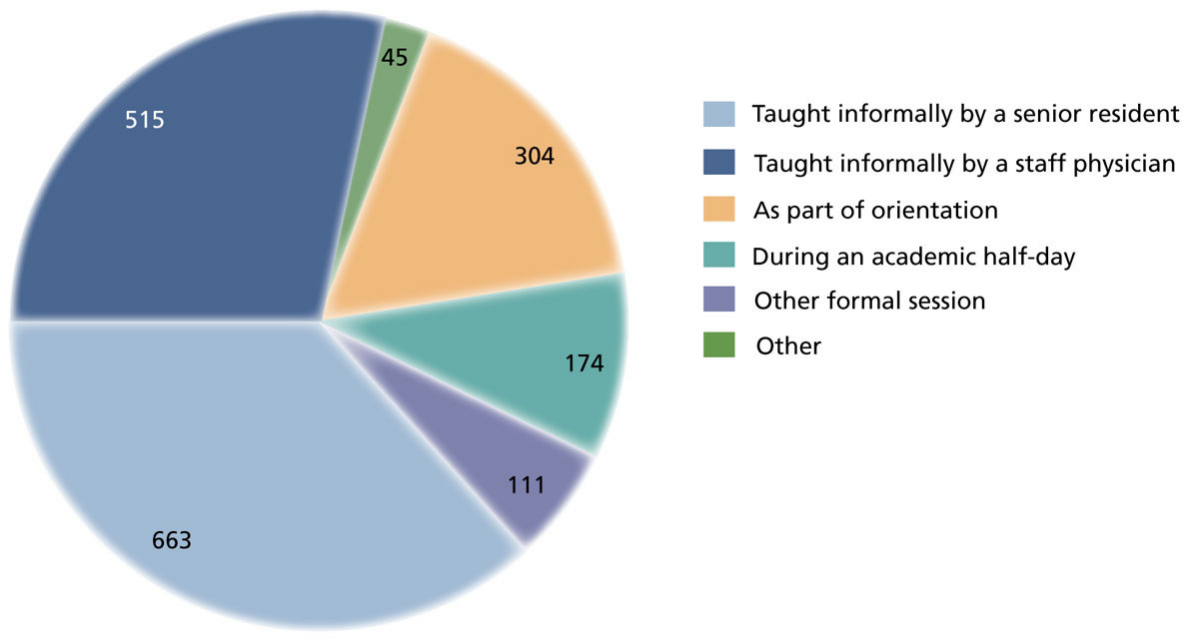
**Frequency of training in patient handover.** Residents were asked to choose all responses that would describe how they had been trained in skills relating to the handover of patient care. Numbers indicate number of responses (n = 830).

The handover training that was received was provided in a variety of formats (Figure 3). The vast majority of training was informal, delivered either by staff physicians or by senior residents. Of those who had received handover training (n = 830), 73% felt that it had improved their ability to provide safe, high-quality care, 20% were unsure, and 7% felt it did not help.

### Formulation of recommendations

Results of the summarized literature and the survey were reviewed by the Advocacy and Policy Committee of CAIR. In consultation with CAIR staff and other CAIR committees, recommendations were developed, reviewed, amended, and approved by consensus by the CAIR Board of Directors.

## Recommendations

In light of these survey data and the literature on resident fatigue, CAIR recommends that the following principles inform the development of policy and standards for resident duty hours in order to ensure patient safety, protect the safety and well-being of all physicians, and provide an optimal educational experience for medical residents [12]:

1. Residents’ hours of work must be managed such that they do not in any way endanger their own or their patients’ health. In particular, limits are required on the number of continuous uninterrupted hours that residents work at a time. In keeping with the evidence base, CAIR urges that all provinces and regions in Canada work towards a system that does not require residents to work more than 16 continuous uninterrupted clinical hours at a time, and that ensures adequate time in between work periods to eliminate the effects of sleep deprivation. This limitation will ensure residents’ ability to provide safe, high-quality patient care while protecting their own personal health and safety.

2. Resident duty hours must be such as to allow for an optimal educational experience. Specifically, trainees’ hours of work must not impair their ability to learn or to train others.

3. Residents must be formally trained in handover skills, i.e., the ability to transfer care appropriately when going off duty.

4. Resident duty hours should be flexible enough to accommodate the specific context of the resident’s role and the service needs on particular rotations.

5. Where a violation of federal or provincial ethical, legislative, or legal standards has occurred, including but not limited to those related to the Canadian Charter of Rights and Freedoms, CAIR calls upon all stakeholders to address and remedy the situation as speedily as possible.

## Discussion

This study does not include residents of Quebec and can thus not be generalized to all of Canada. Discussion among residents, both at CAIR and at the local level, has included a broader understanding and concern for the many issues that surround work hours [13]. These include whether 16 hours is truly a safe limit, or whether some other, shorter number of hours will be found to be optimal; the effect of shorter, possibly more frequent, work periods on resident and physician well-being; the effect of more frequent handovers on patient care; and the effect of chronic fatigue on decision making and learning [14].

One concern is the prolongation of training to enable residents to “see enough cases” and thus obtain adequate clinical exposure and opportunities for learning. Evidence from the literature on sleep deprivation and learning suggests that it is likely that shorter work hours will lead to improved learning and retention, and that residents can be trained “smarter and not harder” [15].

It should be noted that much of the discussion about resident duty hours is centred on traditional academic training centres. However, many residents are now training in non-traditional, community-based, rural or remote centres. It may be entirely appropriate to be on-call for prolonged periods in places where the frequency of active work is low and if there are provisions that enable residents to be relieved of responsibility in the event of an unexpectedly long period of continuous work. Training models will need to accommodate such circumstances.

Although more frequent handovers has been recognized in the literature as a consequence of duty hour restrictions that presents some risk to patient safety, little has been done to improve training in this area. It is remarkable that only 52% of the survey respondents indicated that they had received training in handover skills, while over 73% of those who had received even informal training felt it had improved their ability to provide safe, high-quality care.

## Conclusions

Excessive work hours impair residents’ self-reported ability to provide safe, high-quality care and this has affected over 82% of residents in the current system. This must change in a thoughtful manner that considers the best interest of patients. CAIR endorses the principles of protecting patient safety and resident well-being and safety through shorter shifts, optimizing the educational experience, training in appropriate handover techniques, ensuring flexibility for the specific context, and strictly adhering to existing ethical, legislative, and legal standards in the way we treat resident physicians.

## Authors’ contributions

All authors contributed equally to the preparation of this paper.

## Competing interests

The authors declare that they have no competing interests.

## Supplementary Material

Additional file 1 **Respondent demographics** Residents in all years of training from medical schools in Canada outside of Quebec were invited to participate in the survey. The additional PDF file, titled *Respondent Demographics*, outlines the respondent demographics. Click here for file

## Declarations

Resources and secretariat support for this project was provided by the Royal College.

This article has been published as part of *BMC Medical Education* Volume 14 Supplement 1, 2014: Resident duty hours across borders: an international perspective. The full contents of the supplement are available online at http://www.biomedcentral.com/bmcmededuc/supplements/14/S1. This article was submitted in 2011 and peer reviewed in 2012. Final acceptance for publication as part of this supplement was in 2014.

Publication of this supplement was supported and funded by the Royal College of Physicians and Surgeons of Canada. The funding agency played no role in the design, in the collection, analysis and interpretation of data; in the writing of the manuscripts; and in the decision to submit the manuscript for publication. The articles have been through the journal's standard peer review process for supplements. The Supplement Editors declare that they have no competing interests.
