# Supplementary material for: Resident duty hours in Canada: a survey and national statement
Source: BMC Med Educ. 2014 Dec 11;14(Suppl 1):S9. doi: 10.1186/1472-6920-14-S1-S9 (PMC4304289; doi:10.1186/1472-6920-14-S1-S9)
Supplement: Additional file 1 — Respondent demographics Residents in all years of training from medical schools in Canada outside of Quebec were invited to participate in the survey. The additional PDF file, titled Respondent Demographics, outlines the respondent demographics. [file 1472-6920-14-S1-S9-S1.doc]

## Resident duty hours in Canada: a survey and national statement

## Additional file 2: Respondent demographics

| Characteristic | Response rate (n) |
| --- | --- |
| Residency Year R1  R2  R3  R4  R5  R6  R7  R8+ | 552  464  326  214  153  27  8  10 |
| University University of British Columbia  University of Calgary  University of Alberta  University of Saskatchewan  University of Manitoba  University of Western Ontario  McMaster University  University of Toronto  Northern Ontario School of Medicine (NOSM)  University of Ottawa  Queen’s University  Dalhousie University  Memorial University | 66  152  184  116  124  171  153  328  28  140  101  126  58 |
| Specialty Two-year Family Medicine program  Three-year Family Medicine Enhanced Skills program  Adolescent Medicine  Anatomical Pathology  Anesthesiology  Cardiac Surgery  Cardiology (Adult)  Cardiology (Paediatrics)  Child and Adolescent Psychiatry  Clinical Immunology and Allergy (Adult)  Clinical Immunology and Allergy (Paediatrics)  Clinical Pharmacology  Clinician Investigator Program  Colorectal Surgery  Community Medicine  Critical Care Medicine (Adult)  Critical Care Medicine (Paediatrics)  Dermatology  Developmental Paediatrics  Diagnostic Radiology  Emergency Medicine  Endocrinology and Metabolism (Adult)  Endocrinology and Metabolism (Paediatrics)  Forensic Pathology  Forensic Psychiatry  Gastroenterology (Adult)  Gastroenterology (Paediatrics)  General Pathology  General Surgery  General Surgical Oncology  Geriatric Medicine  Geriatric Psychiatry  Gynecologic Oncology  Gynecologic Reproductive Endocrinology and Infertility  Hematological Pathology  Hematology  Infectious Diseases (Adult)  Infectious Diseases (Paediatrics)  Internal Medicine  Maternal-Fetal Medicine  Medical Biochemistry  Medical Genetics  Medical Microbiology  Medical Oncology  Neonatal-Perinatal Medicine  Nephrology (Adult)  Nephrology (Paediatrics)  Neurology (Adult)  Neurology (Pediatrics)  Neuropathology  Neuroradiology  Neurosurgery  Nuclear Medicine  Obstetrics and Gynecology  Occupational Medicine  Ophthalmology  Orthopaedic Surgery  Otolaryngology  Palliative Medicine  Paediatric Emergency Medicine  Paediatric General Surgery  Paediatric Hematology/Oncology  Paediatric Radiology  Paediatrics  Physical Medicine and Rehabilitation  Plastic Surgery  Psychiatry  Radiation Oncology  Respirology (Adult)  Respirology (Paediatrics)  Rheumatology (Adult)  Rheumatology (Paediatrics)  Thoracic Surgery  Transfusion Medicine  Urology  Vascular Surgery  Other | 351  28  0  30  120  9  8  7  0  1  0  1  8  0  15  8  0  15  0  65  67  4  1  1  0  14  2  9  83  3  1  0  1  1  5  8  3  1  193  2  1  5  7  7  5  9  3  27  8  2  0  19  6  87  0  33  68  21  4  5  1  2  0  104  20  19  145  19  8  0  7  0  0  0  19  0  69 |
